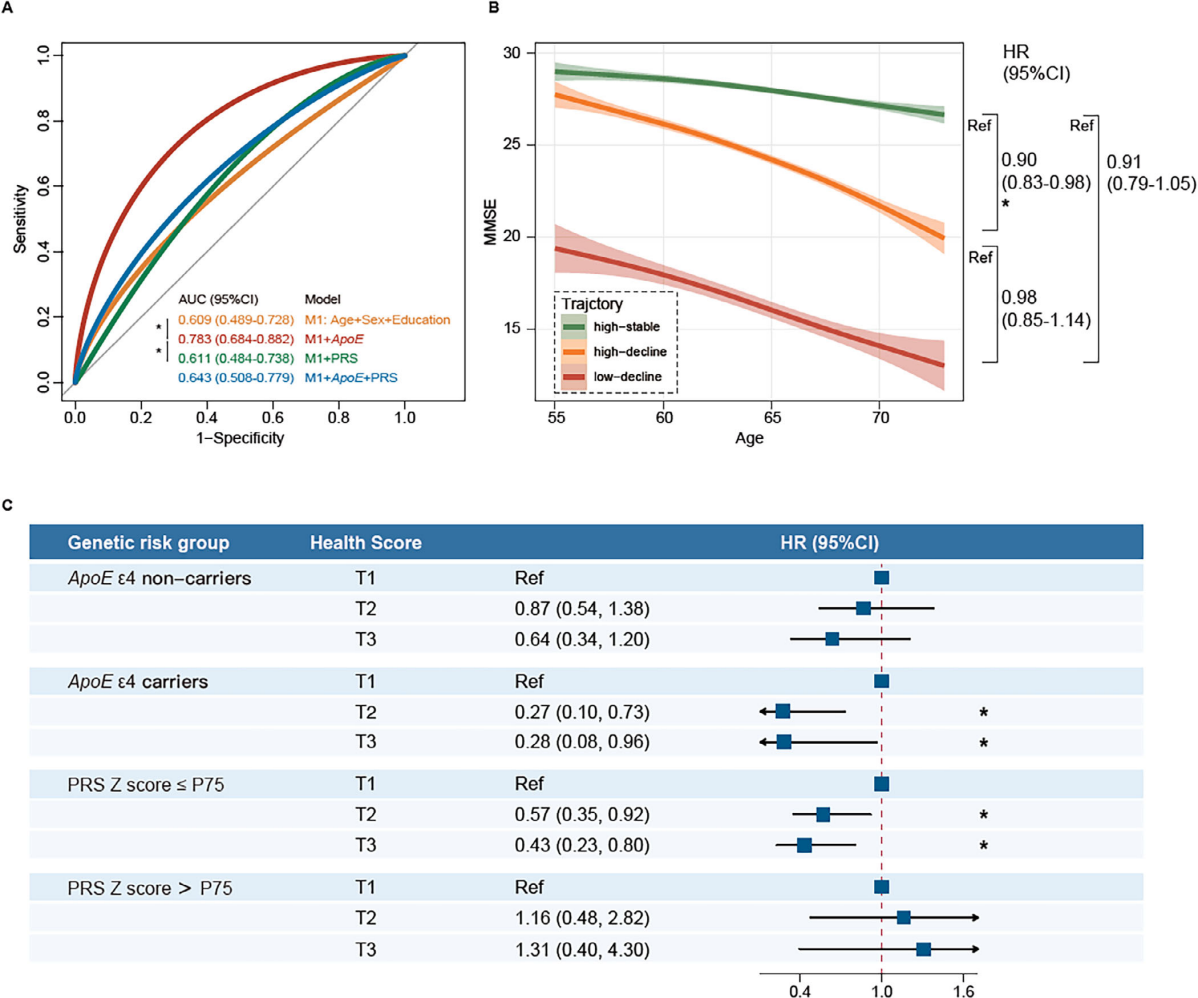# The interplay between genetic susceptibility and modifiable risk factors in the progression of cognitive impairment: A community‐based cohort study

**DOI:** 10.1002/alz70860_101943

**Published:** 2025-12-23

**Authors:** Jialin Li, Jincheng Li, Mei Cui, Yingzhe Wang, Xingdong Chen, Yanfeng Jiang

**Affiliations:** ^1^ State Key Laboratory of Genetic Engineering, Human Phenome Institute, Zhangjiang Fudan International Innovation Center, Fudan University, Shanghai, Shanghai, China; ^2^ Fudan University Taizhou Institute of Health Sciences, Taizhou, Jiangsu, China; ^3^ Huashan Hospital, Fudan University, Shanghai, Shanghai, China

## Abstract

**Background:**

Capturing the trajectory of cognitive decline throughout aging is challenging. Moreover, the interplay between genetic susceptibility to dementia and modifiable risk factors in the onset and progression of cognitive impairment remains unclear. This study investigated whether maintaining favorable lifestyle and environmental factors could mitigate the adverse impact of genetic risk on cognitive decline trajectories and cognitive impairment progression in a community‐based prospective cohort.

**Method:**

A total of 1,526 rural Chinese residents aged 50‐70 years from the Taizhou Imaging Study were visited over four repeated assessments spanning a median of 8 years. Modifiable risk factors (including smoking, sleep, exercise, diet, body mass index, hypertension, hyperglycemia, and hyperlipidemia) were collected via questionnaires and clinical evaluations to calculate a composite health score. Genetic risk was evaluated by *ApoE* genotyping and a polygenic risk score (PRS) (excluding *ApoE*) for cognitive impairment. Latent class trajectory modeling of repeated Mini‐Mental State Examination assessments identified distinct cognitive decline patterns, whereas cognitive impairment progression was defined as a transition from normal cognition to mild cognitive impairment (MCI) or dementia, or from MCI to dementia. Random forest analysis compared the predictive performance of including *ApoE* and PRS, respectively, for cognitive impairment progression, and Cox regression models assessed their joint effects with the health score.

**Result:**

Incorporating *ApoE* ε4 status significantly improved the predictive performance of a traditional model for cognitive impairment progression (area under the curve 0.609 vs. 0.783; DeLong test *p* = 0.029, Figure 1), whereas adding PRS did not. Three cognitive trajectories identified (high‐stable, high‐decline, low‐decline), with a better health score reducing by 10% the risk of transitioning from high‐stable to high‐decline (HR=0.90, 95% CI=0.83‐0.98). Among *ApoE* ε4 carriers and those with low PRS, higher health scores were associated with substantially reduced risks of cognitive impairment progression (HR=0.28 [0.08‐0.96] and 0.43 [0.23‐0.80], respectively).

**Conclusion:**

These findings underscore the protective role of maintaining fewer risk factors for delaying cognitive impairment progression, particularly in *ApoE* ε4 carriers or individuals exhibiting a low additional genetic burden. Targeted lifestyle interventions in such high‐risk populations may substantially reduce the burden of cognitive decline.